# Poly(lactic Acid)–Biochar Biocomposites: Effect of Processing and Filler Content on Rheological, Thermal, and Mechanical Properties

**DOI:** 10.3390/polym12040892

**Published:** 2020-04-12

**Authors:** Rossella Arrigo, Mattia Bartoli, Giulio Malucelli

**Affiliations:** 1 Department of Applied Science and Technology, and local INSTM Unit, Viale Teresa Michel 5, 15121 Alessandria, Italy; rossella.arrigo@polito.it; 2 Department of Applied Science and Technology, C.so Duca degli Abruzzi 24, 10129 Torino, Italy; mattia.bartoli@polito.it

**Keywords:** poly(lactic acid), biochar, melt mixing, solvent casting, rheological behavior, thermal properties

## Abstract

Biocomposites based on poly(lactic acid) (PLA) and biochar (BC) particles derived from spent ground coffee were prepared using two different processing routes, namely melt mixing and solvent casting. The formulated biocomposites were characterized through rheological, thermal, and mechanical analyses, aiming at evaluating the effects of the filler content and of the processing method on their final properties. The rheological characterization demonstrated the effectiveness of both exploited strategies in achieving a good level of filler dispersion within the matrix, notwithstanding the occurrence of a remarkable decrease of the PLA molar mass during the processing at high temperature. Nevertheless, significant alterations of the PLA rheological behavior were observed in the composites obtained by melt mixing. Differential scanning calorimetry (DSC) measurements indicated a remarkable influence of the processing method on the thermal behavior of biocomposites. More specifically, melt mixing caused the appearance of two melting peaks, though the structure of the materials remained almost amorphous; conversely, a significant increase of the crystalline phase content was observed for solvent cast biocomposites containing low amounts of filler that acted as nucleating agents. Finally, thermogravimetric analyses suggested a catalytic effect of BC particles on the degradation of PLA; its biocomposites showed decreased thermal stability as compared with the neat PLA matrix.

## 1. Introduction

The increasing demand for environmentally sustainable products has stimulated a growing research effort in the last years towards the production of new bio-sourced materials, characterized by a lower environmental impact with respect to the traditional fossil-fuel counterparts [1,2,3]. In this context, poly(lactic acid) (PLA) is considered one of the most promising candidates for replacing petrochemical-derived polymers in several fields, including packaging, biomedical, and electronic industries [4,5]. The leading position of PLA is related to its relevant physico-chemical properties, coupled with its bio-sourced origin, compostability, and even biocompatibility [6]. However, in order to obtain high added value PLA-based materials, potentially suitable for high-tech applications, some drawbacks of this biopolymer need to be overcome. In particular, as clearly reported in the literature, its hydrophobicity and lack of modifiable side chain groups, together with its brittleness (elongation at break is below 10%) and low gas barrier properties, limit its complete exploitation for industrial sectors such as automotive, biomedical, electronics, and packaging [7,8]. To accomplish this objective, different strategies have been pursued, including the formulation of blends [9,10], composites [11,12], and nanocomposites [13,14], using a huge variety of either polymeric or non-polymeric dispersed phases. In particular, with the aim to obtain highly sustainable materials, natural and renewable fillers are commonly being selected for the production of PLA-based composites [15,16]. As an example, composites based on PLA and nanocellulose fibrils with high lignin content have been obtained through film casting and hot pressing. The resulting biocomposites showed a significant improvement in mechanical, thermal, and water vapor barrier properties, owing to the effective coupling between PLA and the fillers embedded at nanoscale level [17]. Tesfaye et al. explored the possibility to recycle PLA biocomposites containing different amounts of silk nanocrystals, by subjecting the materials to a repetitive extrusion process [18]; interestingly, a remarkable stabilizing action of the embedded nanocrystals against PLA degradation occurring during multiple processing cycles was observed.

Recently, biochar (BC) has attracted increasing interest as far as its utilization as filler for polymer-based composites, either thermoplastics or thermosets, is concerned [19,20]. BC is usually obtained from the pyrolysis of agricultural and forestry wastes [21], and its structure can be tuned depending on the pyrolysis conditions; in particular, by adjusting the temperature and the oxygen flow, the degree of internal porosity and the presence of different functional groups anchored to the surface can be tailored [22]. The interest towards BC lies in its intriguing properties, such as high chemical and thermal stability, great electric properties, and very large surface area [23], combined with its cost effectiveness and low environmental impact [24]. For instance, electrically conductive composites based on ultra high molecular weight polyethylene and BC derived from bamboo charcoal were formulated through high-speed mechanical mixing and the hot-pressing method [25]. These composites, characterized by a peculiar segregated morphology, exhibited a low electrical percolation threshold and superior thermal stability with respect to the neat matrix. Das et al. formulated polypropylene-based systems containing different amounts of BC produced from landfill pine wood waste [26]. A beneficial effect of BC on the matrix flame behavior was documented; more specifically, the peak of heat release rate and smoke production were remarkable reduced as a result of the BC addition. Besides, the char layer formed after combustion was proven to be effective in enhancing the insulation properties of the formulated composites. Interestingly, the BC derived from the carbonization of starch-based packaging material was melt mixed with post-consumer poly(ethylene terephthalate), to produce a composite filament suitable for 3D printing applications [27]. The introduction of BC particles improved the processability of the matrix, giving rise to composite systems with improved mechanical and thermal properties with respect to the neat polymer matrix.

Generally speaking, biochar is currently exploited as a cheap, functional material, already employed for several applications (i.e., as an adsorber in functional clothing, as storage for volatile nutrients, as energy storage in batteries, and as an insulating material in the building industry, among others) and undoubtedly it is a way for recovering wastes at the end of life, offering them a new added value [28].

Few reports dealing with the formulation of BC-containing composites based on biopolymers are present in the literature [29,30]. For instance, biodegradable poly(butylene adipate-*co*-terephtalate) was melt compounded with BC derived from agriculture commodities to produce composites potentially suitable for food packaging applications [31]. BC addition was found to enhance the thermo-mechanical properties of the polymer matrix, notwithstanding its hydrophobicity. Additionally, the presence of BC particles from bamboo charcoal was demonstrated to be effective in stabilizing the PLA matrix against UV irradiation.

In this work, PLA-based biocomposites containing BC particles derived from spent ground coffee were formulated through two different processing methods, namely melt mixing and solvent casting. BC was introduced within the polymer matrix at different contents, ranging from 1 wt.% to 7.5 wt.%, aiming at evaluating the effect of the processing route and of the BC loading on the ultimate properties of the resulting biocomposites. The dispersion of the embedded BC particles and the extent of the established polymer–filler interactions were assessed through morphological and rheological analyses; besides, the thermal and mechanical behavior of the biocomposites was investigated, assessing the possible influence of the processing on the PLA intrinsic characteristics. Finally, the thermal stability of biocomposites was evaluated and compared to that of neat PLA.

## 2. Materials and Methods 

### 2.1. Materials

Two different PLA matrices were used, PLA 3251D and PLA 4042D, both supplied by IngeoTM Natural Natureworks (Minnesota, MN, USA). PLA 3251D is an injection molding grade and has a melt flow index of 13 g/10 min (210 °C, 2.16 kg); conversely, PLA 4042D is suitable for film applications and shows a melt flow index of 6 g/10 min (210 °C, 2.16 kg).

A waste brewed coffee powder (Caffè Vergnano, Italy) was used as starting material for the production of biochar (BC). In order to remove the leftover chemicals in waste coffee powder after brewing, the sampled waste brewed coffee powder was washed many times with demineralized water. The sample was further centrifuged and filtered and the obtained waste brewed coffee powder was then dried in an oven at 90 °C for 10 h. The pyrolysis of the material was subsequently performed at 700 °C for 1 h in a nitrogen atmosphere (gas flow: 120 mL/min). The heating ramp rate of the tubular furnace was set at 5 °C/min; after the pyrolysis step, the material was manually ground in a mortar, achieving an average size of 10 μm. Further characteristics of the obtained BC particles are reported in [32].

### 2.2. Processing

PLA and PLA-based composites containing 1, 2.5, 5, and 7.5 wt.% of BC were processed through two different methods, namely melt mixing and solvent casting. PLA 3251D was used to formulate solvent cast composites, owing to its lower average molar mass and enhanced solubility in the selected solvent as compared with PLA 4042D.

In melt mixing (MM), the BC powder was compounded with the PLA4042D matrix using a Brabender Plastograph mixer and operating at 170 °C, 100 rpm for 5 min. Neat PLA was subjected to the same processing. Specimens for the various characterization were obtained by means of a compression molding step, using a laboratory press (Collin Teach Line 200T, Maitenbeth, Germany), working at 170 °C, under a pressure of 100 bar for 2 min. Prior to the processing, PLA and BC were vacuum dried overnight at 70 °C. The melt mixed materials were designed as PLA+XBC_MM, where X indicates the BC loading (wt.%).

In the solvent casting (SC) process, PLA 3251D pellets were dissolved in chloroform with vigorous stirring at room temperature. BC was added after the complete dissolution of PLA and the obtained solutions were stirred for 5 h. The solutions were then cast onto a glass Petri dish and allowed to dry for about 24 h at room temperature. The resulting solvent cast films were placed in a vacuum oven at 40 °C for three days in order to remove all remaining solvent. The solvent cast systems were designed as PLA+XBC_SC (X accounts for wt.% BC loading) 

### 2.3. Characterization Techniques

Rheological measurements were performed using an ARES (TA Instrument, New Castle, DE, USA) strain-controlled rheometer in parallel plate geometry (plate diameter: 25 mm). Strain sweep tests were carried out at 170 °C and ω = 1 rad/s. The complex viscosity and storage and loss moduli were measured performing frequency scans from 10^−1^ to 10^2^ rad/s at 170 °C, under a nitrogen atmosphere. The strain amplitude was selected for each sample in order to fall in the linear viscoelastic region. Time sweep tests were carried out at in air at 170 °C, *ω* = 1 rad/s, *γ* = 10%, and for 3600 s.

Microstructures were observed using a LEO-1450VP scanning electron microscope (SEM, Zeiss, Ramsey, NJ, USA) (beam voltage: 20 kV) on the cross-sections of the investigated samples fractured in liquid nitrogen.

Differential scanning calorimetry (DSC) analyses were carried out using a QA1000 TA Instrument apparatus (Waters Lc, Milford, MA, USA). All the experiments were performed under dry N_2_ gas (20 mL/min) using samples of around 8 mg in sealed aluminum pans. All the materials were subjected to the following cycle:

- First heating up from 30 to 200 °C at 10 °C/min;
- Cooling down from 200 to 0 °C at 10 °C/min;
- Second heating up from 0 to 180 °C at 10 °C/min.

All the thermal parameters were evaluated on the second heating scan, erasing the previous thermal history and evaluating T_g_ (glass transition temperature), T_cc_ (cold crystallization temperature), T_m_ (melting temperature), and X_c_ (crystallinity degree) in controlled conditions. X_c_ was calculated as the ratio between the heat of fusion of the sample (ΔH = ΔH_m_ − ΔH_cc_, ΔH_m_ and ΔH_cc_ being the specific melting and cold crystallization enthalpies, respectively), considering only the polymer fraction, and the heat of fusion of a 100% crystalline PLA (93 J/g [33]).

Thermogravimetric analyses (TGA) were performed using a Pyris1TGA apparatus (Perkin Elmer, Shelton, CT, USA) (experimental error: ±0.5 wt%, ±1 °C). Samples (about 10 mg) were placed in alumina pans and runs were carried out in the range of 50–600 °C, with a heating rate of 10 °C/min, under N_2_ flow (35 mL/min). T_5%_, T_10%_ (i.e., the temperatures at which 5% or 10% weight loss, respectively, occurs), and T_max_ values (i.e., the temperatures corresponding to the peaks appearing in derivative - dTG - curves) were calculated; besides, the final residue at 600 °C was measured.

Tensile tests were performed using an Instron^®^ 5966 dynamometer (Norwood, MA, USA). The measurements were carried out at two different crosshead speeds: 1 mm/min until 0.2% of deformation is reached, and then 100 mm/min up to sample breakage. At least five specimens for each system were tested and the were results averaged.

## 3. Results and Discussion

### 3.1. Rheological Behavior

**Figure 1:**
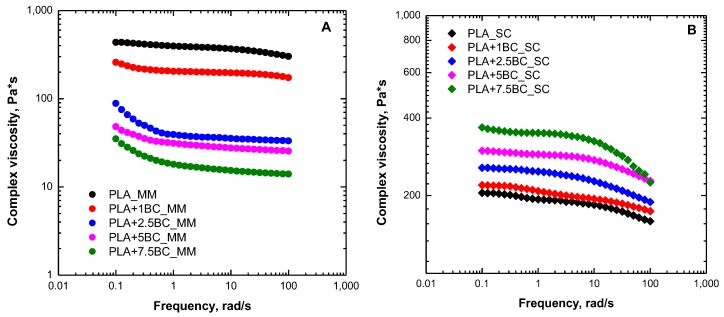
Complex viscosity as a function of frequency for neat poly(lactic acid) (PLA) and biochar (BC)-containing composites obtained through melt mixing (MM) (**A**) and solvent casting (SC) (**B**).

The linear viscoelastic behavior of neat PLA and all formulated composites was evaluated through oscillatory measurements; in Figure 1A,B, the trends of the complex viscosity as a function of frequency for BC-containing composites obtained through melt mixing and solvent casting, respectively, are reported. As far as melt mixed composites are concerned, a progressive decrease of the complex viscosity values with increasing the BC loading can be observed, as compared with the neat PLA matrix. This finding has already been reported in the literature for similar PLA-based composites and attributed to some thermal degradation phenomena occurring in PLA during processing at high temperatures [34]. Specifically, degradation processes, including radical, hydrolysis, and/or transesterification reactions, cause a decrease of the PLA molar mass, resulting in a consequent reduction of the viscosity. However, although all melt mixed composites show reduced viscosity with respect to the neat PLA matrix, it is worth noting that the Newtonian behavior exhibited by pure PLA tends to progressively disappear as the BC loading in the composites increases. In fact, a yield stress behavior can be observed in the low frequency region for all composites, and this behavior becomes more pronounced for systems containing higher amounts of filler. Generally, the appearance of a yield stress behavior is related to the limitation of the macromolecules relaxation owing to the restriction of the chain motion, as a result of the establishment of strong polymer–filler or filler–filler interactions [35]. In the case of BC-containing composites, it can be inferred that, as the BC content increases, a slowing down of the PLA chain mobility occurs, owing to some interactions taking place between the polymer matrix and the embedded fillers.

Looking at the results of the solvent cast systems, a different scenario can be observed. In particular, a progressive increase of the complex viscosity values with respect to those of the neat PLA matrix is noticed, as the filler loading increases. However, different to what was observed in the case of melt mixed composites, the presence of BC particles does not induce the appearance of a stress yield behavior, and BC-containing systems exhibit a frequency-dependence of the viscosity curves very similar to that of neat PLA. In other words, the presence of BC filler causes a mere vertical shift of the complex viscosity curve towards higher values, without actually modifying the relaxation spectrum of PLA macromolecules. Usually, this behavior is observed in polymer-based composites characterized by a low level of polymer–filler interactions, in which the embedded particles are not able to significantly modify the macromolecules dynamics [36]. As a result, the dispersed particles do not induce appreciable rheological alterations and the rheological response of the composites is governed by the matrix; the obtainment of progressively higher viscosity values as a function of the filler content is attributed to higher deformations experienced by the interstitial polymer entrapped between contiguous solid filler particles [37].

**Figure 2:**
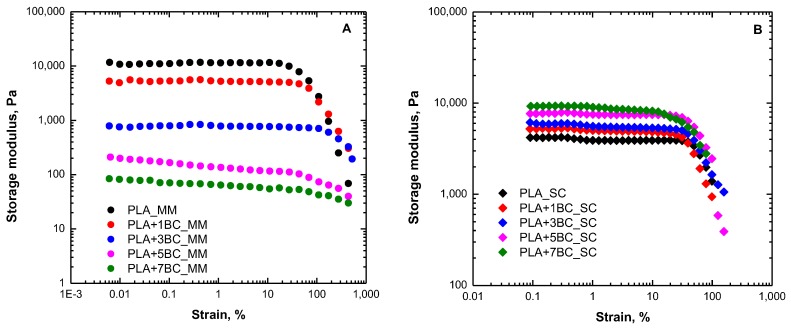
Results of large amplitude oscillatory shear (LAOS) tests for neat PLA and BC-containing composites obtained through melt mixing (**A**) and solvent casting (**B**).

Further important information about the interactions existing in polymer-based composites can be derived from the analysis of the rheological response under large deformations; Figure 2A,B show the results of large amplitude oscillatory shear (LAOS) measurements, in terms of variation of the dynamic storage modulus (G′) as a function of the applied strain amplitude. All investigated systems exhibit a plateau in the low strain region, followed by a decrease of the G′ values. The beginning of the G′ drop is an indication of the transition from linear to non-linear viscoelastic regime and is the result of the preferential alignment of the polymer macromolecules along the flow direction [38]. The results reported in Figure 2 confirm the behavior already noticed through frequency sweep tests; specifically, for melt mixed composites, a progressive decrease of G′ values can be observed as the BC content rises. Conversely, for solvent cast systems, higher modulus values are achieved for composites containing increasing filler amounts. Additionally, for melt mixed composites, an increase of the critical strain at the beginning of the non-linear viscoelastic regime can be noted as a function of BC content, and for PLA+7.5BC_MM, the linear region covers the whole investigated strain amplitude range, indicating that the rheological behavior of composites under large deformations is governed by PLA. At variance, in solvent cast composites, a different trend can be observed as, in this case, the linear viscoelastic range slightly decreases for the systems containing higher filler amounts. 

**Figure 3:**
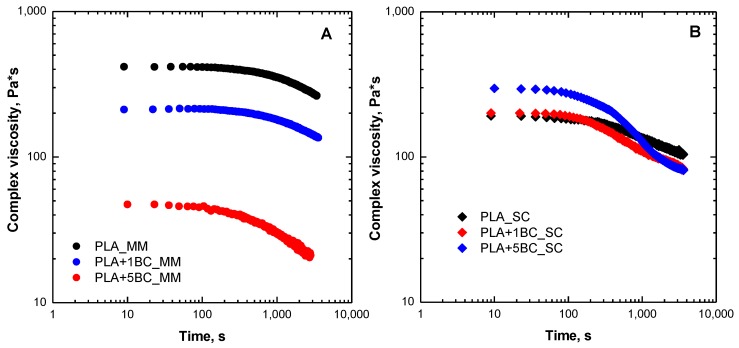
Time evolution of the complex viscosity at 170 °C for neat PLA and BC-containing composites obtained through melt mixing (**A**) and solvent casting (**B**).

To gain a better understanding of the thermally-activated reactions leading to a decrease of the PLA molar mass and to verify the possible effect of BC on the degradation of the matrix in the melt state, the temporal evolution of complex viscosity was evaluated through time sweep measurements at 170 °C in air, for both series of composites. In Figure 3A,B, the trends of viscosity for melt mixed and solvent cast composites containing 1 and 5 wt.% of BC are compared to those of neat counterparts. A progressive decrease of the material viscosity is clearly observable as a function of time also for neat PLA, although the viscosity reduction rate is significantly enhanced by the presence of BC. The observed viscosity drop is more severe for melt mixed composites, which have already been subjected to degradation during the processing performed at a high temperature; furthermore, for these systems, the rate of viscosity decrease is almost unaffected by the filler loading. Conversely, for solvent cast materials, the decrease of viscosity is less pronounced and both the beginning of the viscosity reduction and the rate of viscosity decrease are sensitive to the BC loading.

### 3.2. Morphology 

**Figure 4:**
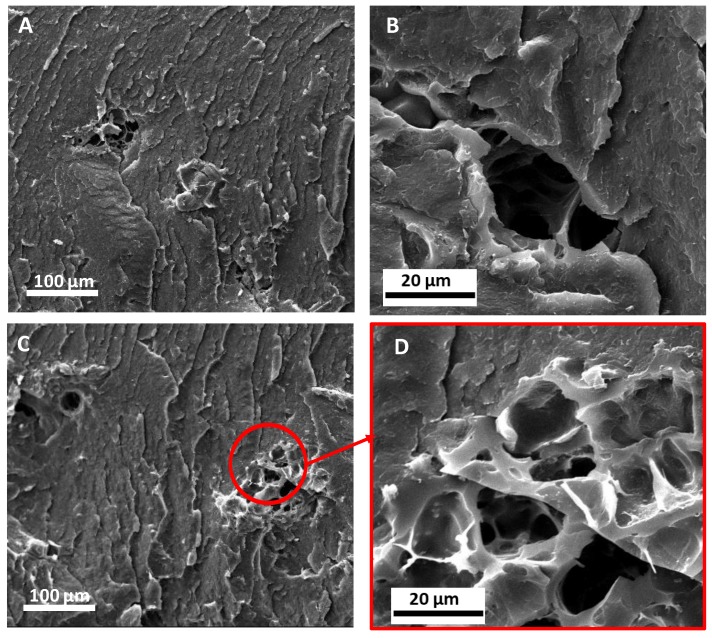
Scanning electron microscope (SEM) micrographs of PLA+7.5BC_MM (**A**,**B**) and PLA+7.5BC_SC (**C**,**D**) at different magnifications.

Some typical SEM micrographs of the fractured surface of composites containing 7.5 wt.% of BC obtained through melt mixing and solvent casting are shown in Figure 4. A homogeneous dispersion of BC particles was achieved for both exploited processing methods, and the embedded fillers do not exhibit aggregation phenomena. Nevertheless, in the case of melt mixed composites, the BC particles’ average size seems to be lower than that observed for solvent cast systems. This phenomenon can likely be ascribed to the intense shear stresses that BC underwent during melt mixing, as during the processing, a partial disruption of particles may occur [39]. This finding can explain the different rheological behavior showed by melt mixed composites with respect to the solvent cast ones, as the size decrease of the BC particles could cause an increase of the polymer/filler interfacial area with a consequent amplification of the interactions between BC and PLA macromolecular chains.

### 3.3. Thermal Properties

**Figure 5:**
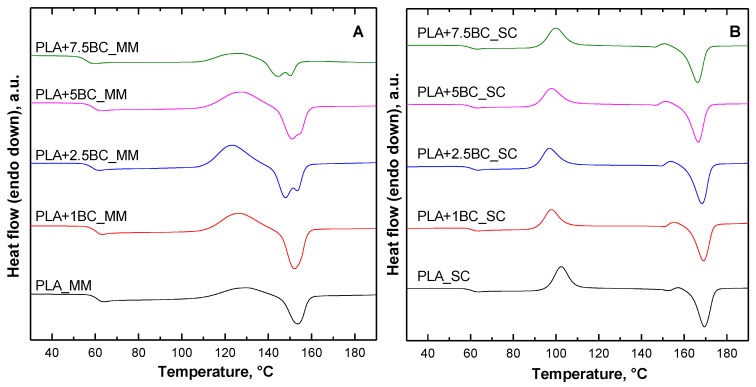
Differential scanning calorimetry (DSC) thermograms recorded during the second heating scan for neat PLA and BC-containing composites obtained through melt mixing (**A**) and solvent casting (**B**).

Figure 5 shows the DSC thermograms recorded during the second heating scan for all investigated systems; the main properties resulting from the selected thermal cycle are listed in Table 1.

As far as melt mixed systems are concerned, the presence of BC causes a slight decrease of T_g_ and T_cc_ values as compared with the neat matrix, indicating an enhanced mobility of PLA macromolecules owing to the molar mass decrease resulting from degradation phenomena occurring during processing. Additionally, beyond 1 wt.% of BC loading, a double melting peak appears. However, the total content of the crystalline phase in melt mixed samples remains almost unchanged as compared with neat PLA, and the materials exhibit an amorphous structure.

Looking at the results shown by solvent cast systems, lower T_g_, T_cc_, and T_m_ values with respect to the neat matrix are observed, suggesting an enhanced crystallization capability. In addition, a remarkable increase of the crystallinity degree can be noticed for composites containing up to 2.5 wt.% of BC; a further increase of the filler content does not favor the crystallization process, and for PLA+7.5BC_SC, only a slight increase of X_c_ as compared with the neat PLA was achieved. The observed behavior can be explained considering two concurrent opposite phenomena exerted by the embedded BC particles: a nucleating effect and an interference on the crystallization process of PLA macromolecules. At low BC loadings, the first effect is prominent and the composites show improved crystallization ability; conversely, for higher amounts of BC, the presence of disperse fillers hampers the crystallization process of polymer macromolecules, and a lower content of crystalline phase is formed.

### 3.4. Thermal Stability

**Figure 6:**
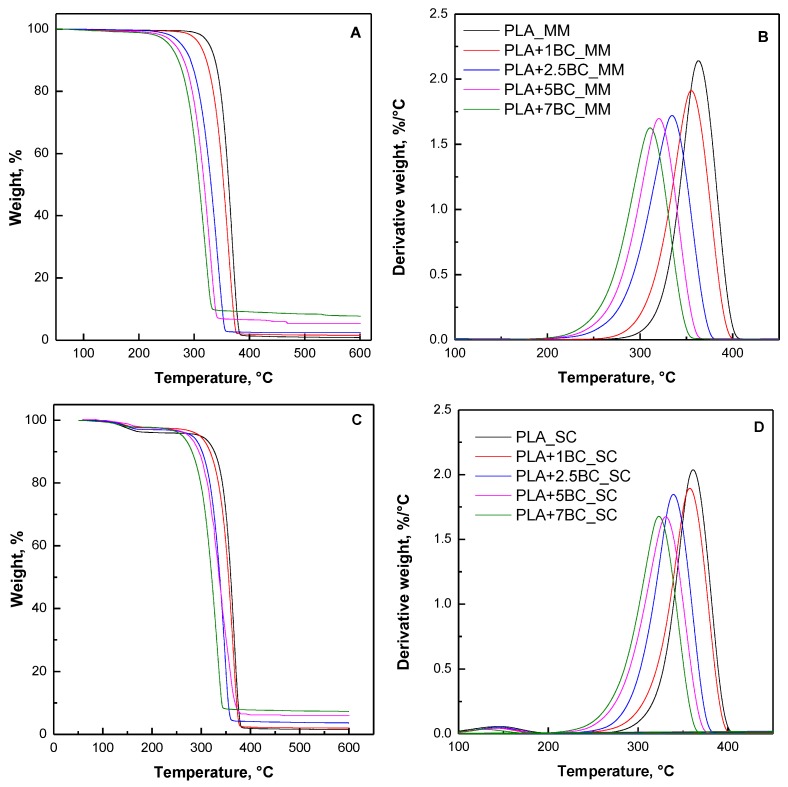
Thermogravimetric (TG) (**A**,**C**) and dTG (**B**,**D**) curves for neat PLA and its composites obtained through melt mixing (**A**,**B**) and solvent casting (**C**,**D**).

Figure 6 reports TG and dTG curves obtained under nitrogen atmosphere for neat PLA and composites; furthermore, Table 2 collects T_5%_, T_10%_, T_Max_, and the final residues at 600 °C in both air and nitrogen atmospheres. In nitrogen, the degradation of the composites occurs in a single degradation step; the weight loss observed for solvent cast systems in the temperature range of 90–180 °C can be ascribed to the removal of the residual solvent in the films [40]. The presence of BC particles caused in both series of composites an anticipation of the degradation phenomena, especially for melt mixed systems. In fact, a progressive decrease of T_5%_ and T_10%_ values can be noticed as a function of the filler content, as well as a gradual shifting towards lower values of the maximum degradation temperature. A detrimental effect of BC particles derived from different sources on the thermal and thermo-oxidative stability of PLA and other bio-polyesters matrices has already been reported in the literature and was attributed to the catalytic effect of potassium, usually contained in BC, on the decomposition of the polymer matrix [31,41]. The presence of potassium in the biochar used in this work has been verified through EDX elemental analysis; the results are reported in Table 3. Therefore, the progressive increase of the potassium content with the increase of BC loading is likely to cause a worsening of the thermal stability of the biocomposites. However, both hydrolytic and “back-bite” reaction mechanisms [42] may take place during the thermal degradation of PLA, owing to residual hydroxyl functionality present on the biochar surface [43].

### 3.5. Mechanical Properties

**Figure 7:**
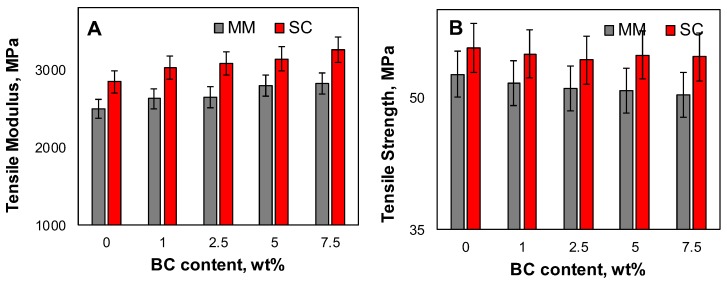
Tensile modulus (**A**) and tensile strength (**B**) for all investigated systems.

The mechanical properties of the PLA-based systems were evaluated through tensile tests; the obtained mechanical properties, namely tensile modulus and tensile strength, are plotted in Figure 7A,B as a function of the BC content. For both melt mixed and solvent cast materials, tensile modulus values increase with the increasing BC loading, confirming the homogeneous dispersion of the embedded filler within the polymer matrix. However, the tensile strength of BC-containing systems is slightly decreased as compared with the neat PLA matrices; this finding can be associated with the premature failure of the biocomposites, owing to the presence of voids in the porous structure of BC particles.

## 4. Conclusions

Two different processing methods, that is, melt mixing and solvent casting, were exploited for preparing PLA-based biocomposites containing BC particles obtained from pyrolysis of spent coffee grounds. The rheological results suggested that melt mixing method is more effective in dispersing BC particles owing to the high shear force that systems experienced during processing, though the high temperature, at which the processing is performed, caused the occurrence of degradation phenomena in PLA, leading to a severe reduction of the polymer molar mass. Conversely, the solvent casting method, carried out at room temperature, preserved PLA from degradation, but the weak dispersing stresses involved in this processing did not promote the establishment of strong polymer–filler interactions, owing to the bigger size of BC and its sponge-like structure. Furthermore, the selected processing route significantly affected the thermal behavior of formulated materials; in fact, melt mixed systems showed an amorphous structure, while solvent casting composites exhibited crystalline phases, owing to the nucleating effect of the embedded BC particles. Finally, irrespective of the processing strategy, an improvement of the tensile modulus was documented for all biocomposites, as compared with neat PLA.

From an overall point of view, the obtained results clearly showed that the ultimate properties of the PLA/BC biocomposites can be profitably modulated by selecting the content of BC and the suitable processing method, which are able to govern the morphology of the materials, as well as their rheological and mechanical behavior.

All these findings may suggest the suitability of BC for replacing some common fillers (such as carbon black, talc, and CaCO_3_) in the preparation of polymer biocomposites with tailored properties, useful for packaging, automotive, and electronic applications. Besides, the low production cost of this carbon filler, together with the possibility of deriving it from different waste sources, clearly represents an added value toward the circular economy approach. The combination of a waste-derived filler together with a biopolymer is thus a very effective tool for the improvement of materials sustainability.

## Figures and Tables

**Table 1:** Main thermal properties collected during the second heating scan for all investigated systems. PLA, poly(lactic acid); BC, biochar; MM, melt mixing; SC, solvent casting.

	$T_{g}$ [°C]	$T_{cc}$ [°C]	$T_{m_{1}}$ [°C]	$T_{m_{2}}$ [°C]	ΔH [J/g]	$X_{c}\;[\%]$	$T_{g}$ [°C]	$T_{cc}$ [°C]	$T_{m_{1}}$ [°C]	ΔH [J/g]	$X_{c}\;[\%]$
	**MM**						**SC**				
PLA	60.7	129.3	-	153.4	1.2	0	61.1	102.5	169.5	13.0	13.9
PLA+1BC	60.4	127.2	-	152.2	3.7	0	60.9	97.8	169.0	21.4	23.2
PLA+2.5BC	59.9	123.9	148	153.4	2.3	0	61.2	96.9	168.3	24.9	29.7
PLA+5BC	59.4	128.3	151.1	152.8	1.6	0	60.4	97.7	166.7	20.2	22.9
PLA+7.5BC	56.1	126.6	144.8	150.2	0.8	0	59.9	99.8	166.3	14.2	16.5

**Table 2:** Thermogravimetric analysis (TGA) results in N2 for neat PLA and BC-containing composites.

Sample	T_5%_ [°C]	T_10%_ [°C]	T_max_ [°C]	Residue at 600 °C [%]	T_5%_ [°C]	T_10%_ [°C]	T_max_ [°C]	Residue at 600 °C [%]
	**MM**				**SC**			
PLA	324.3	335.9	366.9	0.70	300.4	326.5	366.0	0.50
PLA+1BC	310.0	322.2	361.9	1.35	295.0	317.5	363.8	2.00
PLA+2.5BC	277.2	293.6	342.1	2.36	282.1	301.4	347.6	3.16
PLA+5BC	284.0	300.1	343.9	5.51	272.4	291.7	342.6	5.30
PLA+7.5BC	254.8	271.9	320.2	7.41	263.4	281.3	332.0	7.82

**Table 3:** Elemental composition of used BC.

Element	Composition (wt.%)
C	74.0
K	21.6
Ca	4.4
